# Promoting CO_2_ methanation via ligand-stabilized metal oxide clusters as hydrogen-donating motifs

**DOI:** 10.1038/s41467-020-20004-7

**Published:** 2020-12-03

**Authors:** Yuhang Li, Aoni Xu, Yanwei Lum, Xue Wang, Sung-Fu Hung, Bin Chen, Ziyun Wang, Yi Xu, Fengwang Li, Jehad Abed, Jianan Erick Huang, Armin Sedighian Rasouli, Joshua Wicks, Laxmi Kishore Sagar, Tao Peng, Alexander H. Ip, David Sinton, Hao Jiang, Chunzhong Li, Edward H. Sargent

**Affiliations:** 1 Department of Electrical and Computer Engineering, University of Toronto, Toronto, ON M5S 1A4 Canada; 2 Key Laboratory for Ultrafine Materials of Ministry of Education, Shanghai Engineering Research Center of Hierarchical Nanomaterials, School of Materials Science and Engineering, East China University of Science and Technology, Shanghai, 200237 China; 3 Department of Mechanical and Industrial Engineering, University of Toronto, Toronto, ON M5S 3G8 Canada; 4 Department of Materials Science and Engineering, University of Toronto, Toronto, ON M5S 3E4 Canada; 5 School of Chemical Engineering, East China University of Science and Technology, Shanghai, 200237 China

**Keywords:** Catalytic mechanisms, Heterogeneous catalysis, Electrocatalysis

## Abstract

Electroreduction uses renewable energy to upgrade carbon dioxide to value-added chemicals and fuels. Renewable methane synthesized using such a route stands to be readily deployed using existing infrastructure for the distribution and utilization of natural gas. Here we design a suite of ligand-stabilized metal oxide clusters and find that these modulate carbon dioxide reduction pathways on a copper catalyst, enabling thereby a record activity for methane electroproduction. Density functional theory calculations show adsorbed hydrogen donation from clusters to copper active sites for the *CO hydrogenation pathway towards *CHO. We promote this effect via control over cluster size and composition and demonstrate the effect on metal oxides including cobalt(II), molybdenum(VI), tungsten(VI), nickel(II) and palladium(II) oxides. We report a carbon dioxide-to-methane faradaic efficiency of 60% at a partial current density to methane of 135 milliampere per square centimetre. We showcase operation over 18 h that retains a faradaic efficiency exceeding 55%.

## Abstract (web summary)

Electroreduction uses renewable energy to upgrade carbon dioxide to value-added chemicals and fuels. Here, the authors design a suite of ligand-stabilized metal oxide clusters to modulate the reduction pathways on a copper catalyst, enabling record activity for CO_2_-to-methane conversion.

## Introduction

The electrochemical reduction of carbon dioxide (CO_2_) enables the storage of intermittent renewable energy in the form of chemical bonds^1,2^. CO_2_ emissions then become a valuable feedstock in the production of chemical fuels, enabling closing of the carbon cycle^3–5^. Methane (CH_4_) especially benefits from an existing widely-deployed infrastructure for its storage, distribution and utilization^6–8^.

This motivates the need for practical electrolyzers which convert CO_2_ to CH_4_ at high rates and energy efficiency^9,10^. Along the way to this goal, further progress is required in the electrocatalysts which facilitate the conversion chemistry^11–20^. By judicious adjustment of CO_2_ partial pressure, copper (Cu) catalysts have attained a 48% faradaic efficiency (FE) to CH_4_ (ref. ^14^). Fivefold twinned Cu nanowires have achieved a CH_4_ FE of 55% due to a high-density of edge sites available on twin boundaries^15^.

Until now, such systems have operated with good FEs of >50% only at modest current densities of <50 milliampere per square centimetre (mA cm^−2^), below the level needed according to technoeconomic analyses^21^. Electrolysis in systems where CO_2_ is fed in the gas phase overcomes mass transport limitations and as a result is operational at impressive current densities (>100 mA cm^−2^); however, this produces high local pH conditions, promoting the formation of C_2+_ products instead of CH_4_ (refs. ^22–24^).

With the goal of seeking the rational design of more efficient CH_4_ electrocatalysts, we began by examining the factors that influence the CO_2_ methanation pathway. We reasoned that, in flow cell systems, a high local pH results in a lowered driving force for pH-dependent water reduction, and reduces the availability of adsorbed hydrogen (*H) to Cu. Specifically, this militates against the protonation (transfer of *H) of *CO to generate *CHO^25,26^.

To remedy this, we sought to enhance the kinetics of the proton-transfer step: we would strive to increase H_2_O reduction to provide the necessary *H, but without significantly altering the binding energy of intermediates interacting with Cu active sites.

In this work, we design and synthesize a suite of ligand-stabilized cobalt oxide (CoO) nanoclusters. We show that judicious selection of CoO nanocluster size enables control over surface *H coverage for tuneable modulation of CO_2_ methanation. Optimization of CoO nanocluster size (2.5 nm) enables the cooperative system to achieve a methane FE of 60% at an operating current density of 225 mA cm^−2^ and continuous operation for over 18 h in a flow cell system, without significant loss in performance.

## Results

### Density functional theory calculations

We began by exploring CO_2_ methanation pathways using computational investigations. Recent studies^27–29^ of CO_2_ reduction pathways on Cu surface show that the *CO intermediate undergoes either a hydrogenation step (Eq. (1)) toward CH_4_, or a dimerization step (Eq. (2)) toward C_2+_ products.

1 $$\ast {\mathrm{CO}} + \ast {\mathrm{H}} \to \ast {\mathrm{CHO}}$$

2 $$\ast {\mathrm{CO}} + \ast {\mathrm{CO}} \to \ast {\mathrm{OCCO}}$$

Steering the CO_2_ reduction pathway toward CH_4_ requires enhancing the local *H availability to promote hydrogenation of the *CO intermediate over the dimerization (C–C coupling) step.

Coupling the Cu surface with *H generating motifs would provide a means to tune *H availability and thereby direct the reaction toward CH_4_. These motifs reduce H_2_O to produce *H, which will hydrogenate *CO on neighbouring Cu atoms to *CHO. We also compared the *CO hydrogenate energies on pure Cu via the H_2_O-assisting *Heyrovsky* mechanism vs. the *H transfer mechanism^30,31^ (0.63 vs. 0.43 eV, Supplementary Fig. 1): the *H transfer mechanism is more likely from a thermodynamic analysis.

**Fig. 1:**
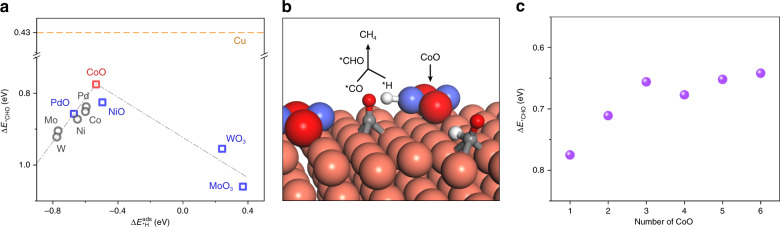
Density functional theory calculations of carbon dioxide methanation. **a** Relationship between hydrogenation energy of *CO and surface *H binding energy for pure metal and metal oxide clusters on Cu surface. Red square for CoO, blue square for other oxides and grey circle for metals. The yellow dashed line is Cu baseline. **b** Schematic diagram of CO_2_ to CH_4_ on CoO cluster modified Cu surface. Tangerine for copper, violet for cobalt, red for oxygen, grey for carbon and white for hydrogen. **c** Hydrogenation energy of *CO for different sizes of CoO cluster. We introduced one cluster on the top of a 4-layer (5 × 2) Cu(111) surface.

We therefore introduced into density functional theory (DFT) models a series of motifs (metal and metal oxide) with varying *H binding energy on Cu as the catalyst for CO_2_ reduction reaction (CO_2_RR) and calculated the energetics of *CO hydrogenation by the *H transfer mechanism (see ‘Methods’). The *CO hydrogenation energy for the pure Cu surface is 0.43 eV (Fig. 1a); however, Cu favours the dimerization step toward C_2+_ products when the local *H availability is insufficient^27–29^. We considered a variety of modified Cu surfaces and found a volcano-like relationship between the *CO hydrogenation energies and the *H binding energies (Fig. 1a). Metal (as distinct from metal oxide) motifs possess strong *H binding energy, preventing them from donating *H for *CO hydrogenation. Some high-valence metal oxides (e.g. molybdenum and tungsten trioxides), exhibit a weaker *H binding energy but fail to stabilize *H adsorption near Cu.

The addition of CoO, which has an intermediate *H binding energy, results in the lowest reaction energy for hydrogenation of *CO (Δ*E* = 0.78 eV) among these motifs. This finding suggests that addition of CoO clusters could enhance the local *H availability and enable facile *CO hydrogenation formed on neighbouring Cu atoms and increase the FE toward CH_4_ (Fig. 1b).

We further investigated the influence of the CoO-cluster sizes on CH_4_ selectivity (Fig. 1c). The results show that a larger CoO-cluster size increases *CO hydrogenation activity; however, too large CoO clusters also enhance hydrogen evolution reaction (HER) activity which competes with CO_2_RR: indeed, calculations point to the need to optimize the cluster diameter.

### Synthesis and characterization of CoO clusters

We sought experimentally to create such a system, beginning by synthesizing CoO clusters of four different sizes, 1.5, 2.1, 2.5 and 2.8 nm (see ‘Methods’). Prior studies have suggested that ligands may protect the oxidized clusters from reduction to the metallic phase during the synthesis^32–34^. The CoO clusters dispersed in solution are light purple; however, if the ligand is absent in the synthesis, this results in a black solution (Supplementary Fig. 2).

**Fig. 2:**
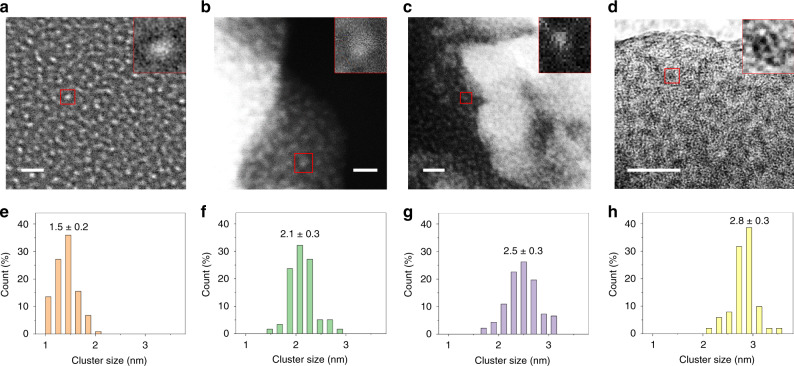
Preparation and characterization of cobalt(II) oxide clusters. **a**–**d** Transmission electron micrograph images and **e**–**h** corresponding size distribution of CoO clusters. The white light dots in (**a**–**c**) and dark black dots in (**d**) are the CoO clusters. Insets in (**a**–**d**) are the enlarged images showing the individual CoO cluster in red square. The scale bars in (**a**, **b**) and (**c**, **d**) are 5 and 10 nm, respectively.

Seeking to characterize the dispersion and size distribution of the four CoO clusters, we performed scanning transmission electron microscopy (STEM, Fig. 2a–c) and high-resolution TEM (HRTEM, Fig. 2d). The homogeneous bright features in Fig. 2a–c and dark dots in Fig. 2d are the CoO clusters with different sizes of 1.5 ± 0.2, 2.1 ± 0.3, 2.5 ± 0.3 and 2.8 ± 0.3 nm, respectively (Fig. 2e–h).

### Integrated CoO/Cu system

Using Nafion solution as a binding agent (see ‘Methods’), we spray-coated the CoO clusters onto a Cu sputtered polytetrafluoroethylene (Cu/PTFE) catalyst^14,22^ for CO_2_RR. To assess the impact of the clusters on the hydrogen evolution reaction (HER) activity of Cu, we performed linear sweep voltammetry in a flow cell system with Ar gas using 1 M KHCO_3_ electrolyte. This was carried out with CoO clusters of mean size 2.5 nm integrated with the Cu/PTFE catalyst (denoted CoO-2.5 nm/Cu/PTFE). As controls, bare Cu/PTFE and Cu/PTFE integrated with metallic Co nanoparticles (denoted Co metal/Cu/PTFE) were tested as well (Supplementary Fig. 3). We observed HER activity to follow the sequence Co metal/Cu/PTFE > CoO-2.5 nm/Cu/PTFE > Cu/PTFE. This enhancement in HER indicates that the ligand does not completely block all the *H adsorption sites present on the CoO clusters.

**Fig. 3:**
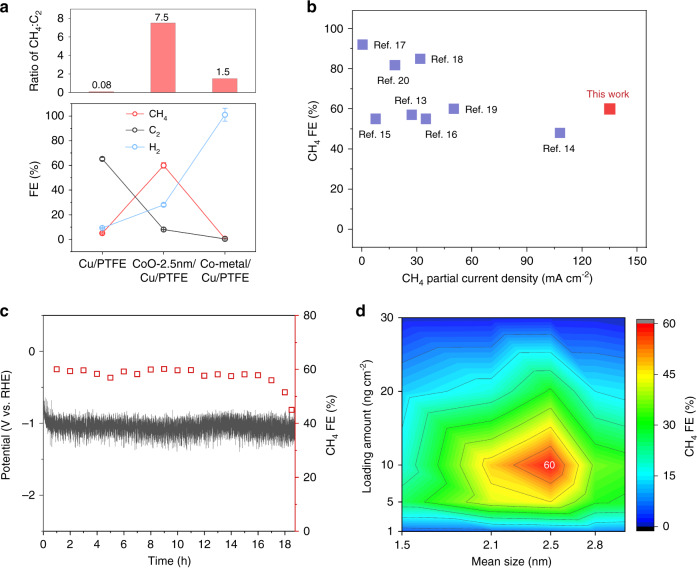
Electrocatalytic performance of electrocatalysts. **a** Product distribution of CoO/Cu/PTFE, Co metal/Cu/PTFE and bare Cu/PTFE at the −1.1 V vs. RHE. Red circle for methane, blue circle for hydrogen and grey circle for C_2_ products. **b** CO_2_-to-CH_4_ performance of CoO/Cu/PTFE in comparison with recent reports^13–20^. Blue square for the results in literature. **c** Stability test of CO_2_ methanation during 18.5 h of electrolysis under the current density of 225 mA cm^−2^. Red square for methane FE and grey line for potential curve. **d** CH_4_ FEs of ligand-stabilized CoO clusters with different sizes and loading contents on Cu/PTFE under an operating current density of 225 mA cm^−2^ in a flow cell using 1 M KHCO_3_ electrolyte.

We then used the same flow cell system with CO_2_ gas and 1 M KHCO_3_ to assess the CO_2_RR performance (Supplementary Table 1). At an applied potential of −1.1 V versus reversible hydrogen electrode (V vs. RHE), CoO-2.5 nm/Cu/PTFE, Co metal/Cu/PTFE, and bare Cu/PTFE attained CH_4_ FEs of 60%, 0.6%, and 5%, respectively (Fig. 3a). The CoO-2.5 nm/Cu/PTFE catalyst achieved a CH_4_ partial current density of 135 mA cm^−2^; however, the bare Cu/PTFE and Co metal/Cu/PTFE controls reach only 12.5 and 1.35 mA cm^−2^, respectively.

From the product distributions, it is clear that the increased CH_4_ FE comes at the expense of C_2+_ products such as ethylene and ethanol on CoO-2.5 nm/Cu/PTFE. The ratio of CH_4_:C_2+_ increases from 0.08 on bare Cu/PTFE to 7.5 on CoO-2.5 nm/Cu/PTFE (Fig. 3a). For Co metal/Cu/PTFE, the current is channelled almost entirely toward HER (~100% FE): this corresponds to the situation where *H binds strongly to the motif and is unable to promote the hydrogenation of *CO on neighbouring Cu atoms.

We noted that previous work^23^ has shown that modifying *H binding energy with hydroxide motifs allowing steering of post C–C coupling pathways toward ethanol instead of ethylene on Cu/PTFE; however, these experiments were performed under higher pH conditions (1 M KOH) and at lower applied potentials (−0.6 V vs. RHE) compared to this work. Herein, the neutral electrolyte and high work potential (−1.1 V vs. RHE) favour C_1_ products^10,27^, and *H binding energy has a greater effect on the branching points between methane formation and C–C coupling in CO_2_RR.

To exclude the influence of the poly(methacrylic acid) ligand on CO_2_RR activity, we spray-coated the ligand onto bare Cu/PTFE without CoO clusters. We found negligible differences in product selectivity for these samples vs. bare Cu/PTFE (Supplementary Table 1), allowing us to conclude that the ligand does not have a direct influence on CO_2_ reduction pathways.

We found the high CH_4_ partial current density of 135 mA cm^−2^ (Fig. 3b) and CO_2_-to-CH_4_ conversion rate of 0.17 μmol cm^−2^ s^−1^ of CoO-2.5 nm/Cu/PTFE, which improve by a factor of 2.6x relative to the best previous reports having a CH_4_ FE higher than 50% (Supplementary Table 2). The catalyst system reported herein achieved a half-cell energy efficiency of 27% in neutral medium (Supplementary Note 1), which is among the best previously reported catalysts with current densities above 50 mA cm^−2^ (Supplementary Table 2)^13–20^.

We then tested the stability of the CoO-2.5 nm/Cu/PTFE catalyst system. The system achieved CO_2_-to-CH_4_ generation over 18 h at a current density of 225 mA cm^−2^ with a CH_4_ FE above 55% (Fig. 3c). After 18 h, the CH_4_ FE decreased to below 55%. After the catalyst was washed and dried, CO_2_RR was resumed with the same catalyst: the CH_4_ FE returned to the original value of 60% (Supplementary Fig. 4), suggesting that the decrease of CH_4_ FE is not caused by catalyst degradation but could be due to flooding or carbonate formation on the PTFE substrate^22,35^.

We also investigated the CO_2_-to-CH_4_ performance as a function of CoO-cluster size. CoO-2.5 nm with a loading content of 10 ng cm^−2^ on Cu/PTFE catalyst (an estimated coverage of 1%, see Supplementary Note 2) gives the highest CH_4_ selectivity of 60% (Fig. 3d). An increase in loading content regardless of cluster size leads to an increase in H_2_ evolution activity which diminishes the CH_4_ FE (Supplementary Table 3).

When examining the selectivity of these electrocatalysts as functions of CoO-cluster size and loading content, we draw two conclusions within the size of 1–3 nm and loading content of 1–30 ng cm^−2^ range: (I) cluster size effects are present, and (II) there exists a maximum in CH_4_ selectivity with a CoO-cluster size of 2.5 nm and 10 ng cm^−2^ on Cu/PTFE in this work.

### Characterization of CoO-cluster-localized catalysts

**Fig. 4:**
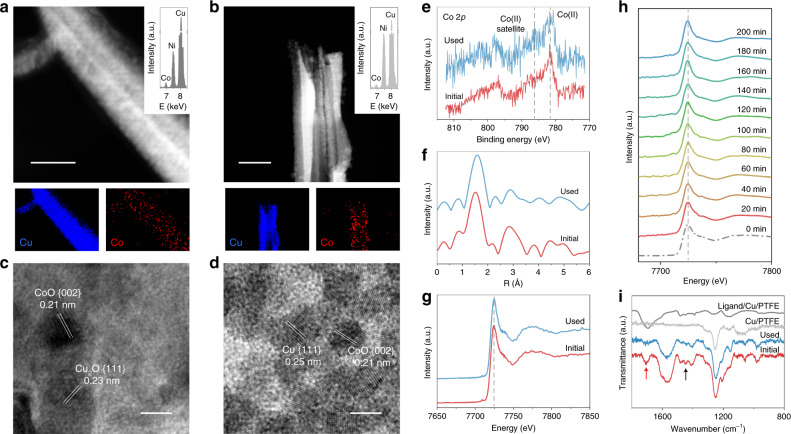
Structural characterization of electrocatalysts. **a**, **b** Dark-field scanning transmission electron micrograph images and corresponding energy-dispersive X-ray spectroscopy mapping for Cu (blue dot) and Co (red dot). **c**, **d** High-resolution transmission electron micrograph images of the CoO-2.5 nm/Cu/PTFE catalysts before and after stability test. The scale bars in (**a**, **b**) and (**c**, **d**) are 200 and 5 nm, respectively. **e** Co 2*p* X-ray photoelectron spectroscopy spectra. **f** The *k*^2^-weighted Fourier-transform spectra from extended X-ray absorption fine structure and **g** the normalized X-ray absorption near-edge structure spectra at the Co *K*-edge of the CoO-2.5 nm/Cu/PTFE catalysts before and after stability test. Red and blue lines for before and after stability test, respectively. **h** *Operando* Co *K*-edge of X-ray absorption fine structure of CoO-2.5 nm/Cu/PTFE catalyst under an operating current density of 225 mA cm^−2^ in a flow cell using 1 M KHCO_3_ electrolyte. **i** Fourier-transform infrared spectra of the CoO-2.5 nm/Cu/PTFE catalysts before and after stability test, compared with the bare and ligand localized Cu/PTFE. Red and blue lines for CoO-2.5 nm/Cu/PTFE catalyst before and after stability test, respectively; light grey and dark grey lines for Cu/PTFE and ligand/Cu/PTFE controls, respectively. Red and black arrows for –CO– and –COO– in the poly(methacrylic acid) ligand, respectively. a.u. presents arbitrary unit.

To investigate possible catalyst changes in structure and oxidation state following CO_2_RR, we carried out characterization of CoO-2.5 nm/Cu/PTFE before and after the extended electrochemical operation studies, using electron microscopy and X-ray absorption spectroscopy. Scanning electron microscopy (SEM) images of CoO-2.5 nm/Cu/PTFE catalysts demonstrate reconstruction of Cu after CO_2_RR (Supplementary Fig. 5). STEM images with elemental mapping reveal a uniform distribution of Cu and Co in samples before and after stability tests (Fig. 4a, b). HRTEM images show intimate contact between CoO clusters and the Cu/PTFE catalyst (Fig. 4c, d). According to the measured lattice fringes^23^ in the TEM images, the initial slightly oxidized Cu is reduced to its metallic state during CO_2_RR.

The lack of Co related peaks in X-ray diffraction patterns can be attributed to the small size and low loading of CoO clusters (Supplementary Fig. 6). Cu 2*p* high-resolution X-ray photoelectron spectroscopy (XPS) spectra reveal the existence of metallic and oxidized Cu species in the initial catalyst and mostly metallic Cu after CO_2_RR (Supplementary Fig. 7)^23^.

The Co 2*p* spectra show Co(II) in the CoO-2.5 nm/Cu/PTFE catalysts before and after stability testing (Fig. 4e). On the other hand, Co 2*p* spectra in Co metal/Cu/PTFE after the test shows metallic character, which is as expected (Supplementary Fig. 8)^36^. The O 1*s* spectra indicate the presence of metal oxides (Supplementary Fig. 9). Fourier-transformed spectra of the Co *K*-edge extended X-ray adsorption fine structure (EXAFS) of the CoO-2.5 nm/Cu/PTFE catalysts before and after the stability test are in Fig. 4f. The single notable peak, a feature that is at 1.0–2.0 Å, is ascribed to Co–O binding^37^. Figure 4g shows the normalized X-ray absorption near-edge structure (XANES) spectra. The white-line position reflects the Co(II) oxidation state of Co–O bond^37,38^, consistent with the view that the clusters include oxidized CoO.

To investigate whether ligand-stabilized CoO clusters remain as an oxide during CO_2_RR, we carried out *operando* X-ray absorption spectroscopy (XAS) to determine the valence states of Co during CO_2_ electroreduction by focusing on the Co *K*-edge spectra. From these studies, we see that the CoO clusters remain in their oxidized form during electrocatalysis over 3 h at an operating current density of 225 mA cm^−2^ (Fig. 4h). It is in these conditions that the catalyst achieves its best CO_2_-to-CH_4_ FE of 60%. We did not observe a change in the white-line position of Co species throughout electrolysis. Fitting results of the Co–O bond XAS spectra reveal a negligible change of coordination number (~4.5) and bond length (~2.0 Å) for initial vs. *operando* catalysts (Supplementary Table 4). This leads us to the view that the ligand may indeed protect the CoO clusters, allowing them to remain oxidized under the reducing conditions applied during CO_2_RR, at least over the timescales of operation (~3 h) studied herein.

We also performed *operando* Raman spectroscopy for CoO-2.5 nm/Cu/PTFE samples at a current density of 225 mA cm^−2^ over a period of 90 min (Supplementary Fig. 10a). The spectra exhibit peaks in the range of 500–600 cm^−1^, which is assigned to Co–O^39^. Upon application of a reducing potential, these peaks do not diminish in intensity even after 90 min, providing supporting evidence that CoO species are stabilized, over the timescale studied here, against reduction by the ligand. As a control, the *operando* Raman spectroscopy was also carried out on the bare Cu/PTFE sample under similar reaction conditions (Supplementary Fig. 10b). We observe only the presence of small peaks at 530 cm^−1^ related to Cu_2_O^40^, which disappears completely within 10 min of reduction. The Raman spectra for the CoO/Cu/PTFE and bare Cu/PTFE electrode before electrocatalysis (Supplementary Fig. 10c) show peaks assigned to Cu oxide^40^. To exclude the possibility of the Raman signals from CoO on the electronically uncontacted parts in PTFE, we performed *operando* Raman for two more controls: (I) commercial CoO (purchased from Sigma-Aldrich) on Cu/PTFE and (II) ligand-stabilized CoO clusters on Cu/GDL substrate (i.e., sputtered Cu on conductive carbon paper with gas diffusion layer). The Raman results of the first control (Supplementary Fig. 11) show that the Co–O bonds are completely vanished after 10 min of CO_2_ reduction reaction; while the second control (Supplementary Fig. 12) presents similar Raman peaks in the range of 500–600 cm^−1^ compared to those in the Cu/PTFE as substrate, suggesting that CoO remains over the course of the CO_2_ reduction studies herein.

The presence of the ligand in catalysts before and after CO_2_RR is indicated by Fourier-transform infrared spectroscopy analysis, showing that poly(methacrylic acid) ligand is retained during the CO_2_RR (Fig. 4i). In addition, the peak at 1550 cm^−1^ is attributed to the carboxylate groups in the ligand associated with metal ions (here is Co) in a bridging mode^41^.

We noted that stronger-binding organic ligands on Co(II) did not prevent Co metal formation under negative cathodic potentials in prior literature reports^42^; however, in these prior studies, the electrolyte was acidic phosphate buffer (pH 2.2), and the cathodic reaction was hydrogen evolution, compared to the 1 M KHCO_3_ solution (pH 8.9) studied in CO_2_ reduction herein. The local pH is higher during electrochemical CO_2_ reduction, particularly so at high current density (such as 225 mA cm^−2^ when we obtained the best CH_4_ FE)^43^. The stability of Co^δ+^ based catalysts, including Co^δ+^ complexes^44^ and ultra-small/thin cobalt oxides^45,46^, under negative potentials for electrocatalytic CO_2_ reduction, has previously been reported. In a broader context, there are other examples in which metal oxide surface remain during electrochemical CO_2_ reduction, even though the Pourbaix diagram suggests that the metallic phase is thermodynamically stable^47,48^. We propose that the CoO clusters are ligand-stabilized CoO to the point that, under the conditions of pH and applied potential discussed here, the oxidation state and catalytic enhancement can be maintained under the conditions of current and experimental duration reported herein.

We also prepared the molybdenum(VI), tungsten(VI), nickel(II) and palladium(II) oxides clusters as controls (Supplementary Fig. 13), with the ligand stabilization and similarly spray-coated on Cu/PTFE. The microscopy and spectroscopy analyses show that these clusters are oxidized^49–52^ and these catalysts exhibit similar structures to CoO/Cu/PTFE (Supplementary Figs. 14–17). Similar catalytic trends are obtained using these catalysts with their different respective ligand concentrations and cluster loading contents (Supplementary Figs. 18–20 and Tables 5–8). Of the catalysts, CoO-2.5 nm/Cu/PTFE exhibits the highest CH_4_ selectivity.

## Discussion

We developed a cooperative system designed on the principle that local *H availability can provide enhanced CO_2_ methanation at a catalyst/electrolyte interface, with size effects playing a role in localized CoO clusters. The CoO-cluster size is controlled from 1.5 to 2.8 nm and the clusters are reduction-resistant under a cathodic potential, a finding we attribute to stabilization using a poly(methacrylic acid) ligand. These clusters serve to modulate the CO_2_ reduction pathways toward CH_4_ formation. On the CoO/Cu/PTFE catalyst, the reaction energetics are altered to favour CO hydrogenation. The catalysts exhibit a CH_4_ FE of 60% at a current density of 225 mA cm^−2^; these operate over 18 h while retaining initial performance to within 91%.

## Methods

### Catalyst preparation

For CoO clusters, 8.8 mg of cobalt(II) chloride (99.9%, Sigma-Aldrich) and 20 mg of poly(methacrylic acid) ligand^32–34^ were added into a screw-neck glass bottle containing 5 ml of methanol. After the solution becoming uniform by sonication, 0.5 ml of freshly prepared NaBH_4_ solution (5 mg ml^−1^ in methanol) was injected into the solution under vigorous stirring (2000 r.p.m.). The stirring speed of the solution was kept at the same for another 10 min. In the washing process, the precipitate was sonicated to ensure all clusters dispersing uniformly in methanol, and then separated by centrifuge. The precipitate was washed for two times and stored in methanol for further use. For different sizes of CoO clusters, the ligand concentrations are 2, 4, 8 and 16 mg ml^−1^ for synthesize cluster with mean size of 2.8, 2.5, 2.1 and 1.5 nm, respectively. The metal Co nanoparticles were synthesized using the similar process without adding the ligand. For different clusters, we only changed the corresponding chloride salts (tungsten(VI) chloride, 99.9%, Sigma-Aldrich; molybdenum(V) chloride, 99.99%, Sigma-Aldrich; nickel(II) chloride, 99.99%, Sigma-Aldrich; palladium(II) chloride, 99.9%, Sigma-Aldrich) as the precursor and the concentration is the same 0.8 mg ml^−1^ (metal-based). Cu/PTFE electrodes were prepared by sputtering a Cu layer of 200 nm in thickness onto a PTFE membrane (average pore size of 450 nm) using a Cu target (99.99%) at a rate of 1 Å s^−1^. Then, 10 μl of 5% Nafion was dispersed in 1 ml the cluster suspension (0.2 mg ml^−1^ in methanol) and the mixture was under ultrasonication for 30 min. The suspension was deposited on a Cu/PTFE electrode using spray-coating.

### Materials characterization

The morphology of the electrodes was characterized using scanning electron microscopy (SEM, Hitachi S-5200) with a 5-kV beam voltage. Transmission electron microscopy (TEM) and elemental mapping images were collected using a Hitachi HF-3300, at an acceleration voltage of 300 kV, equipped with a Bruker energy-dispersive X-ray spectroscopy (EDX) detector. The acquisition time in the EDX studies was 3 min. The samples for TEM measurements are dip-coated on the carbon-coated nickel grids. Fourier-transform infrared spectroscopy (FTIR) was performed using a Nicolet 6700 FTIR spectrometer. Measurements were carried out using the attenuated total reflection (ATR) mode using the iS50-ATR-FTIR system. Spectra were obtained using 16 scans with a resolution of 4 cm^−1^. Powder X-ray diffraction (XRD) patterns were recorded using a Bruker D8 using Cu-Kα radiation (*λ* = 0.15406 nm). X-ray photoelectron spectroscopy (XPS) was conducted on a PHI 5700 ESCA System using Al Kα X-ray radiation (1486.6 eV) for excitation. Some exposure to air during cell disassembly and the transfer to XPS equipment is expected. *Operando* Raman spectroscopy measurements were carried out using a Renishaw inVia Raman microscope in a modified flow cell (Supplementary Fig. 10d) using a water immersion objective with a 785-nm laser.

XAS measurements were carried out at 9-BM beamline at the advanced photon source (APS) light source (IL, USA). An in-house custom-made electrochemical cell with a three-electrode configuration was used to perform *operando* XAS. A platinum mesh and Ag/AgCl electrode were used as the counter electrode and reference electrode, respectively. The catalyst was prepared on the front side of PTFE and connected electrically by Cu tape while the back side was stuck to a thin Kapton tape. The sample was then mounted on the window of the electrochemical cell so that the back side (PTFE gas diffusion layer) faced the beam and the catalyst (front side) is in direct contact with the electrolyte. The ex situ XAS experiments, including XANES and EXAFS, were conducted on the front side of the samples. Data was collected in fluorescence mode on a passivated implanted planar silicon (PIPS)/Lytle detector placed at an angle of 45°. Several spectra were collected and averaged at each condition to improve the quality of the data and increase the signal to noise ratio. Data post-processing and fitting was performed using the Demeter software package.

### Electrochemical measurements

We used an electrochemical flow cell containing a gas chamber, a cathodic chamber, an anodic chamber and an anion exchange membrane (separating the anodic and cathodic chambers) to study the electrochemical CO_2_ reduction performance. We fixed the working electrode (such as CoO/Cu/PTFE) with a geometric active surface area of 1 cm^2^ and place it between the gas and cathodic chambers. We made the catalysts layer side of the working electrode face to the cathodic chamber. We chose 1 M KHCO_3_ solution as the electrolyte, and used peristaltic pumps to circulate the electrolyte through the cathodic and anodic chambers with a rate of 10 ml min^−1^. We controlled the CO_2_ gas flow rate through the gas chamber as 50 sccm by a gas flow controller. We operated the electrochemical tests on an Autolab PGSTAT204, with a reference Ag/AgCl electrode and a counter Ni foam electrode. We calibrated the potentials to those vs. RHE after *iR*-compensation. We collected the gas products from the end of the gas chamber and analysed them by a gas chromatography (PerkinElmer Clarus 600) with a flame ionization detector (FID) and a thermal conductivity detector (TCD). We evaluated the hydrogen evolution activities of the electrodes in the same flow cell using argon as the only flow gas.

### DFT calculations

We performed density functional theory calculations with the Vienna Ab Initio Simulation Package (VASP) code^53,54^. The exchange-correlation energy was described by Perdew–Burke–Ernzerhof (PBE) functional within the generalized gradient approximation (GGA)^55^. The ionic cores were simplified using the projector augmented wave (PAW) pseudo-potentials^56^. After a series of tests, we set up the cutoff energy of 450 eV. In all calculations, the Hellmann–Feynman forces criteria between atoms were set as 0.02 eV Å^−1^ and the electronic iterations convergence was 10^−5^ eV using the Normal algorithm. A 4-layer (5 × 2) Cu (111) supercell was built to simulate the most stable exposed surface of copper accompanied with a sufficient vacuum gap of 15 Å. Pure metal (Co, Ni, Pd, W and Mo) and metal oxide (CoO, NiO, PdO, WO_3_ and MoO_3_) clusters were added on the fixed positions of Cu (111) surface. Structural optimizations were then performed on all modified slab models. *CO, *CHO and *(CO+H) were considered as key intermediates to simulate the CO_2_-to-methane reaction on catalyst surfaces. During all adsorption calculations, the top two layers were fully relaxed while the other layers were fixed at the tested lattice position. After comparing the hydrogenation activity of Co atom and O atom (Supplementary Fig. 21), we consider Co sites in clusters as the active sites for *CO hydrogenation. The adsorption energies and hydrogenation energies were calculated according to Supplementary Note 3.

## Supplementary information

Supplementary Information

## Data Availability

The data that support the findings of this study are available from the corresponding author upon reasonable request.
