# Supplementary material for: Promoting CO2 methanation via ligand-stabilized metal oxide clusters as hydrogen-donating motifs
Source: Nat Commun. 2020 Dec 3;11:6190. doi: 10.1038/s41467-020-20004-7 (PMC7713075; doi:10.1038/s41467-020-20004-7)
Supplement: Supplementary file 1 — Supplementary Information [file 41467_2020_20004_MOESM1_ESM.pdf]

Supplementary Information

Promoting CO_2 methanation via ligand-stabilized metal oxide clusters as hydrogen-donating motifs

Yuhang Li[1,2,†], Aoni Xu[1,†], Yanwei Lum[1,†], Xue Wang[1], Sung-Fu Hung[1], Bin Chen[1], Ziyun Wang[1], Yi Xu[3], Fengwang Li[1], Jehad Abed[1,4], Jianan Erick Huang[1], Armin Sedighian Rasouli[1], Joshua Wicks[1], Laxmi Kishore Sagar[1], Tao Peng[1], Alexander H. Ip[1], David Sinton[3], Hao Jiang[2,5], Chunzhong Li[2,5*] & Edward H. Sargent[1*]

[1] Department of Electrical and Computer Engineering, University of Toronto, Toronto, Ontario M5S 1A4, Canada

[2] Key Laboratory for Ultrafine Materials of Ministry of Education, Shanghai Engineering Research Center of Hierarchical Nanomaterials, School of Materials Science and Engineering, East China University of Science and Technology, Shanghai 200237, China

[3] Department of Mechanical and Industrial Engineering, University of Toronto, Toronto, Ontario M5S 3G8, Canada

[4] Department of Materials Science and Engineering, University of Toronto, Toronto, Ontario M5S 3E4, Canada

[5] School of Chemical Engineering, East China University of Science and Technology, Shanghai 200237, China

[†] These authors contributed equally: Yuhang Li, Aoni Xu, Yanwei Lum

*Emails: czli@ecust.edu.cn (C.L.) and ted.sargent@utoronto.ca (E.H.S.)

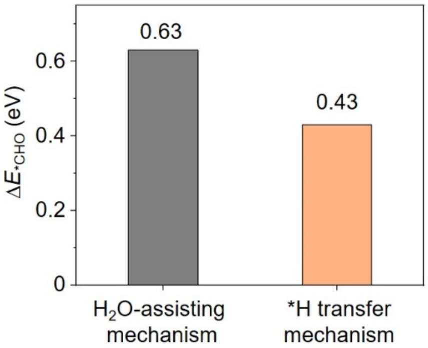

Supplementary Figure 1. *CO hydrogenation energy for pure Cu surface by the H_2O-assisting *Heyrovsky* mechanism and *H transfer mechanism.

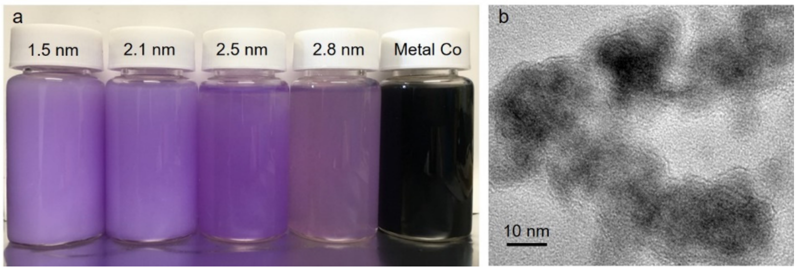

Supplementary Figure 2. (**a**) A photo comparing the ligand stabilized CoO clusters with different sizes and without the ligand. The concentration of all solution is 0.2 mg ml^{-1} (metal-based). (**b**) A typical TEM image of Co nanoparticles (without the ligand) with a mean size of 10 nm.

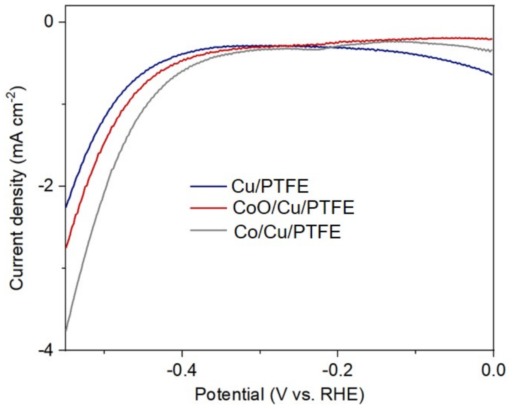

Supplementary Figure 3. Hydrogen evolution reaction polarization curves of different electrodes in Ar-saturated 1 M $KHCO_3$ using flow cell system.

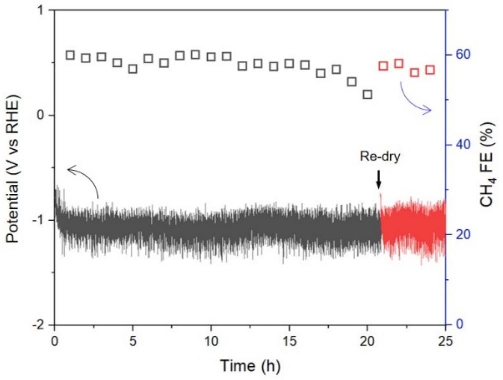

Supplementary Figure 4. Faradaic efficiency of methane (right axis) of CoO-2.5nm/Cu/PTFE catalyst along with corresponding V-t curve (life axis) at a current density of 225 mA cm^{-2}.

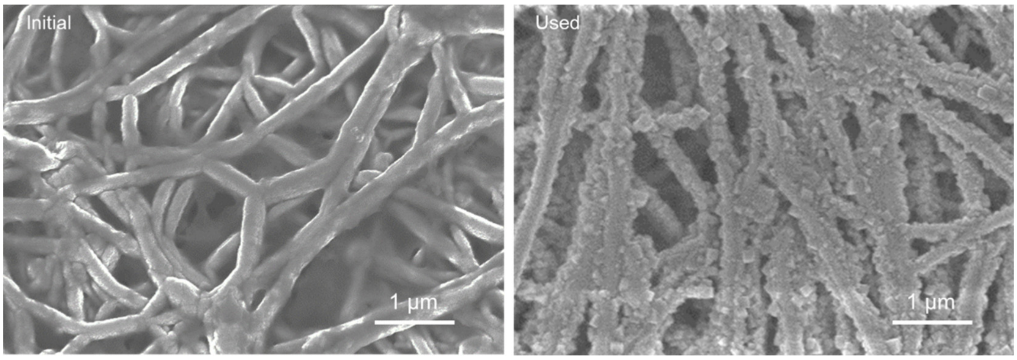

Supplementary Figure 5. Scanning electron microscope images of the CoO-2.5nm/Cu/PTFE catalysts before and after electrocatalytic tests.

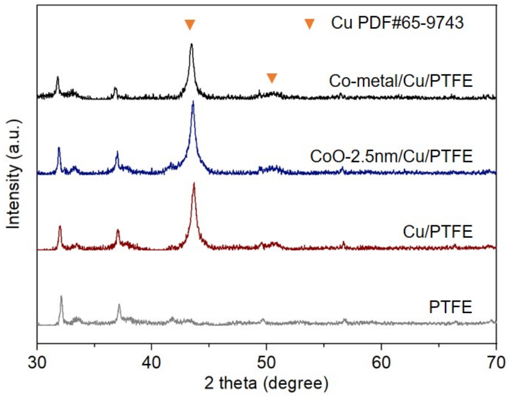

Supplementary Figure 6. XRD patterns for CoO and Co metal localized Cu/PTFE, compared with Cu/PTFE and the bare PTFE substrate.

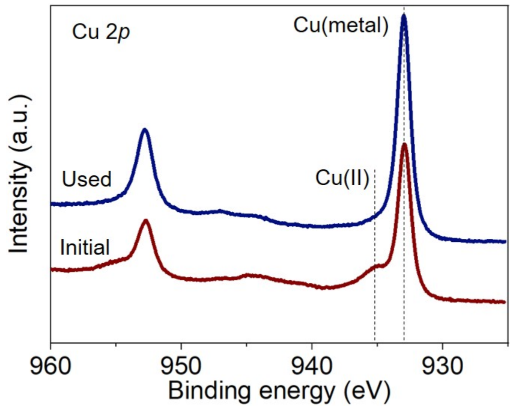

Supplementary Figure 7. High-resolution X-ray photoelectron spectroscopy of Cu 2*p* of the CoO-2.5nm/Cu/PTFE catalysts before and after electrocatalytic tests.

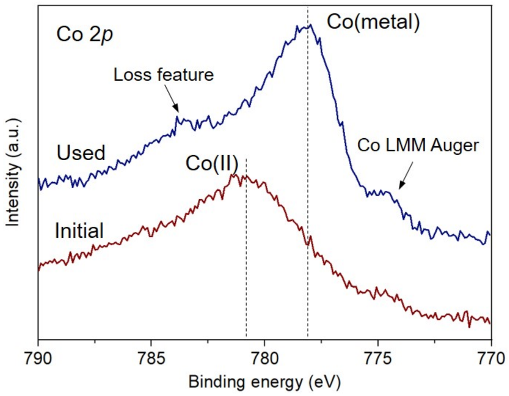

Supplementary Figure 8. High-resolution X-ray photoelectron spectroscopy of Co 2*p* of the Co-metal/Cu/PTFE catalysts before and after electrocatalytic tests.

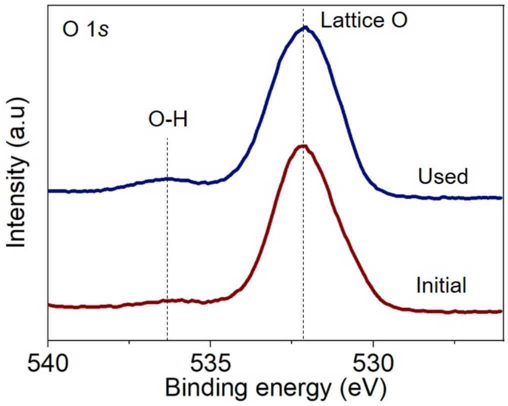

Supplementary Figure 9. High-resolution X-ray photoelectron spectroscopy of O 1*s* of the CoO-2.5nm/Cu/PTFE catalysts before and after electrocatalytic tests.

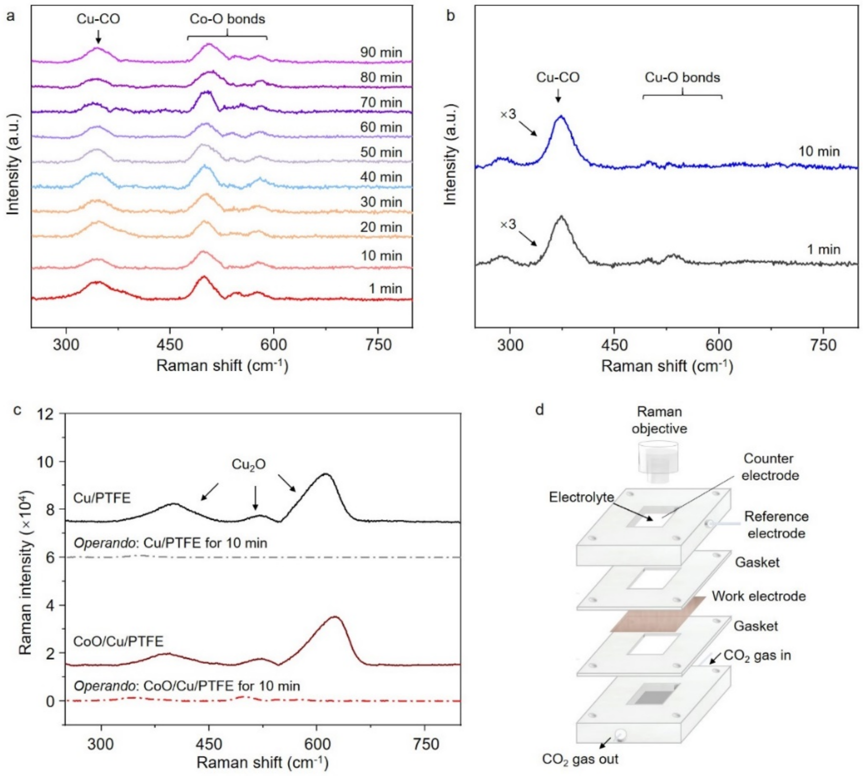

Supplementary Figure 10. (**a**) *Operando* Raman spectra of the CoO/Cu/PTFE and (**b**) Cu/PTFE samples in flow cell with 1 M $KHCO_3$ as the electrolyte at a current density of 225 mA cm^{-2}, after background subtraction. ×3 represents signal magnification. (**c**) Raman spectra of the samples in flow cell with 1 M $KHCO_3$ as the electrolyte before applying negative potentials. The *operando* spectra are listed for comparison (dash lines). (**d**) Schematic illustration of the home-built electrochemical cell for *operando* Raman measurement. Raman measurements were conducted using a Renishaw inVia Raman microscope and a water immersion objective with a 785 nm laser. An Ag/AgCl (3 M KCl) electrode and a Pt wire were used as the reference and counter electrodes, respectively.

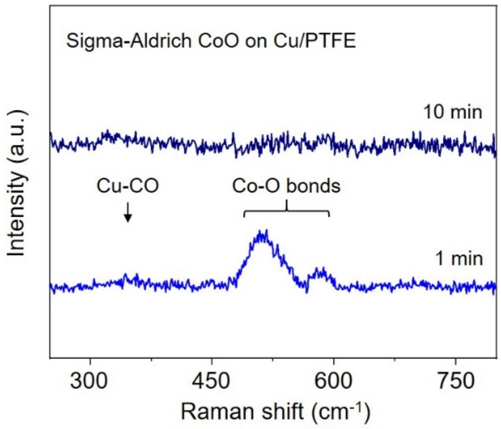

Supplementary Figure 11. *Operando* Raman spectra of commercial CoO powder (purchased from Sigma-Aldrich) on Cu/PTFE in flow cell with 1 M $KHCO_3$ as the electrolyte at a current density of 225 mA cm^{-2}, after background subtraction. We noted that the Cu-CO peaks in the control sample are weak, which corresponds well to the fact that hydrogen evolution (rather than CO_2 reduction) dominates on this catalyst (product FEs in Supplementary Table 1).

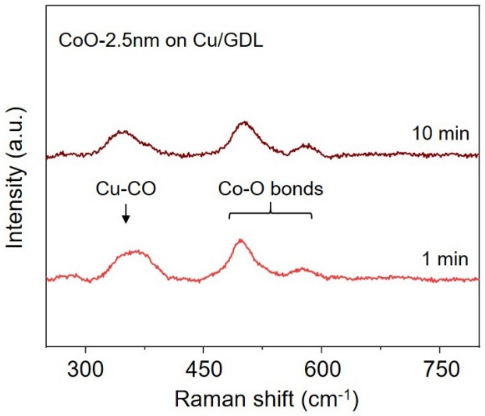

Supplementary Figure 12. *Operando* Raman spectra of the ligand-stabilized CoO clusters on the fully conductive Cu/GDL substrate in flow cell with 1 M $KHCO_3$ as the electrolyte at a current density of 225 mA cm^{-2}, after background subtraction.

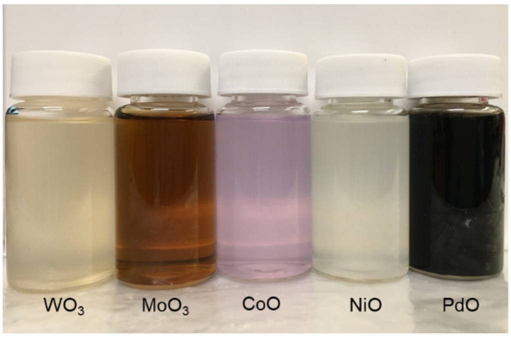

Supplementary Figure 13. A photo comparing the ligand stabilized different oxidized clusters solution with the same concentration of 0.2 mg ml^{-1} (metal-based). The ligand concentration is 4 mg ml^{-1} in synthesis.

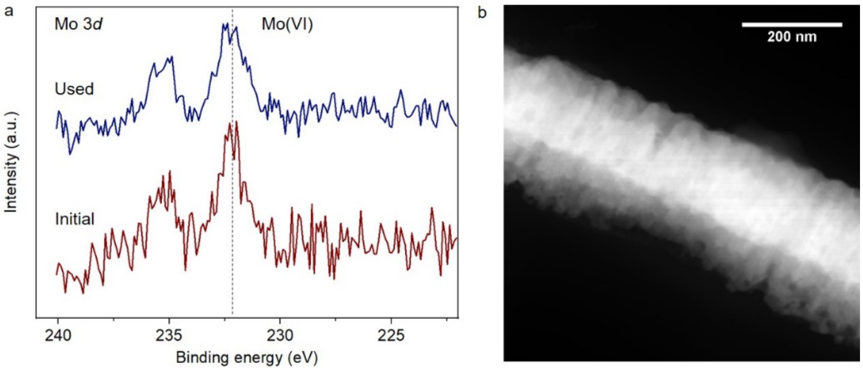

Supplementary Figure 14. (**a**) High-resolution X-ray photoelectron spectroscopy of Mo 3*d* of the MoO_3/Cu/PTFE catalysts with best CH_4 FE of 36% before and after electrocatalytic tests. (**b**) A typical STEM image of the MoO_3/Cu/PTFE catalyst with best CH_4 FE of 36%.

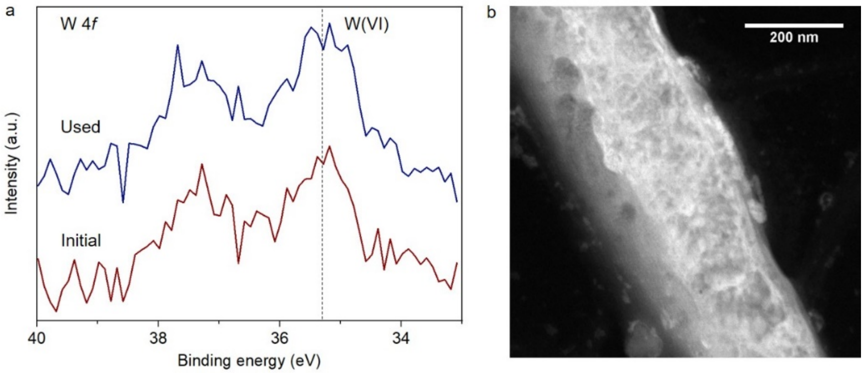

Supplementary Figure 15. (**a**) High-resolution X-ray photoelectron spectroscopy of W 4*f* of the WO_3/Cu/PTFE catalysts with best CH_4 FE of 38% before and after electrocatalytic tests. (**b**) A typical STEM image of the WO_3/Cu/PTFE catalyst with best CH_4 FE of 38%.

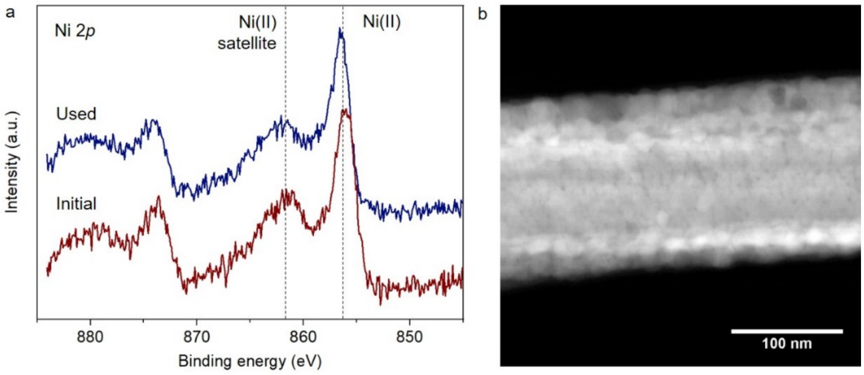

Supplementary Figure 16. (**a**) High-resolution X-ray photoelectron spectroscopy of Ni 2*p* of the NiO/Cu/PTFE catalysts with best CH_4 FE of 50% before and after electrocatalytic tests. (**b**) A typical STEM image of the NiO/Cu/PTFE catalyst with best CH_4 FE of 50%.

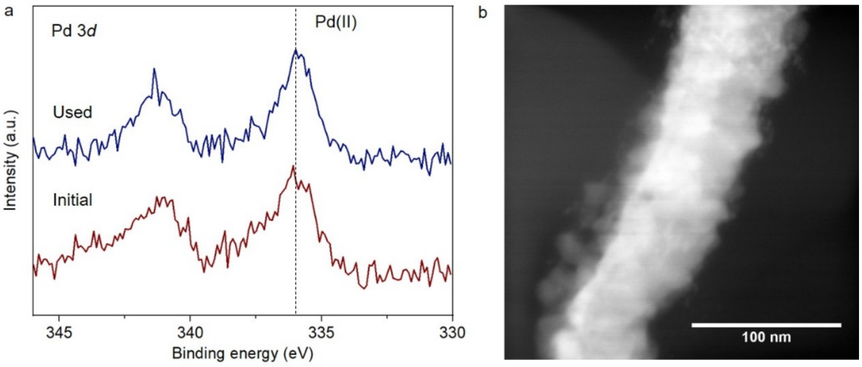

Supplementary Figure 17. (**a**) High-resolution X-ray photoelectron spectroscopy of Pd 3*d* of the PdO/Cu/PTFE catalysts with best CH_4 FE of 47% before and after electrocatalytic tests. (**b**) A typical STEM image of the PdO/Cu/PTFE catalyst with best CH_4 FE of 47%.

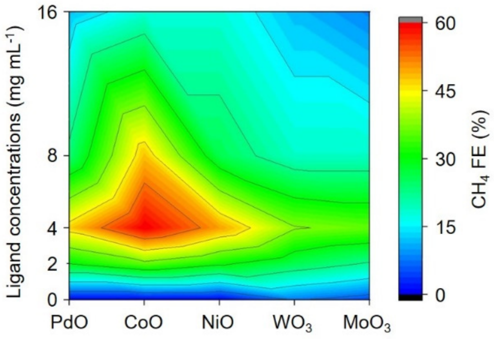

Supplementary Figure 18. CH_4 FEs of different clusters with different ligand concentrations in synthesis and a loading content of 10 ng cm^{-2} on Cu/PTFE under a current density of 225 mA cm^{-2} in a flow cell using 1 M $KHCO_3$ electrolyte.

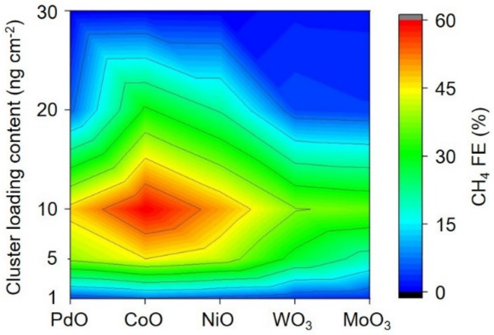

Supplementary Figure 19. CH_4 FEs of different clusters with a ligand concentration of 4 mg mL^{-1} in synthesis and different cluster loading contents on Cu/PTFE under current density of 225 mA cm^{-2} in a flow cell using 1 M $KHCO_3$ electrolyte.

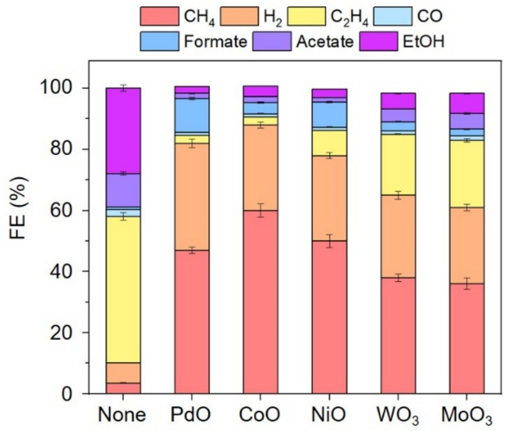

Supplementary Figure 20. Product distribution of the oxidized clusters localized on Cu/PTFE electrode with best CH_4 FEs using 1 M $KHCO_3$ at an operating current density of 225 mA cm^{-2}. The error bars represent the standard deviation from three independent tests.

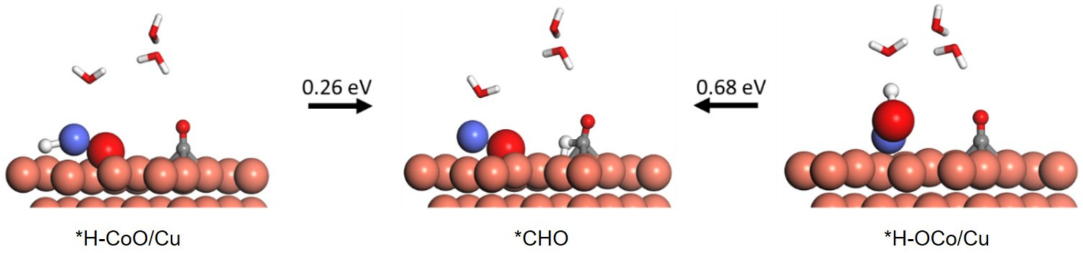

Supplementary Figure 21. Comparison between hydrogenation energies for *H on Co atom and O atom in cluster.

Supplementary Table 1. FEs to various CO_2RR products on different electrodes as a function of working current density.

Notation	J (mA cm^{-2})	E (V vs RHE_{iR})	CH_4 FE (%)	H_2 FE (%)	C_2H_4 FE (%)	CO FE (%)	Formate FE (%)	Acetate FE (%)	EtOH FE (%)
Cu/PTFE	100	-0.95	0.6 ± 0.1	6.2 ± 0.2	43 ± 1.2	8.5 ± 0.3	5.4 ± 0.2	6.9 ± 0.1	24 ± 1.0
	150	-1.01	1.2 ± 0.1	6.4 ± 0.2	45 ± 1.2	4.8 ± 0.2	2 ± 0.1	8.1 ± 0.3	26 ± 1.1
	200	-1.02	1.5 ± 0.1	6.5 ± 0.2	46 ± 1.5	4 ± 0.2	1.8 ± 0.1	8.9 ± 0.3	27 ± 1.1
	225	-1.04	3.5 ± 0.2	6.6 ± 0.1	48 ± 1.2	2.2 ± 0.1	0.8 ± 0.1	11 ± 0.5	28 ± 1.0
	250	-1.14	5 ± 0.5	9.3 ± 0.3	45 ± 1.2	1.5 ± 0.1	0.5 ± 0.1	6.2 ± 0.2	14 ± 0.5
Co-2.5nm/ Cu/PTFE	100	-1.02	15 ± 0.5	18 ± 0.8	26 ± 0.9	4.5 ± 0.2	2.1 ± 0.1	7.8 ± 0.3	22 ± 1.4
	150	-1.09	29 ± 1.2	22 ± 1.0	19 ± 0.7	3.8 ± 0.1	2.6 ± 0.1	6.2 ± 0.3	16 ± 1.0
	200	-1.09	39 ± 1.4	26 ± 0.8	8.6 ± 0.4	2.2 ± 0.1	3.2 ± 0.2	5.1 ± 0.2	11 ± 0.3
	225	-1.11	60 ± 2.2	28 ± 1.2	2.6 ± 0.1	1.1 ± 0.1	3.7 ± 0.2	1.9 ± 0.1	3.5 ± 0.1
	250	-1.22	41 ± 1.6	40 ± 2.0	2.5 ± 0.1	0.8 ± 0.1	8.4 ± 0.3	1.5 ± 0.1	3.4 ± 0.1
Co-metal/ Cu/PTFE	225	-1.11	0.6 ± 0.1	101 ± 5.2	0.2 ± 0.1	0.1 ± 0.01	0.1 ± 0.01	0.1 ± 0.01	0.1 ± 0.01
Commercial Co/Cu/PTFE	225	-1.11	0.5 ± 0.1	99 ± 4.5	0.2 ± 0.1	0.1 ± 0.01	0.1 ± 0.01	0.1 ± 0.01	0.1 ± 0.01
Ligand/ Cu/PTFE	225	-1.04	3.6 ± 0.1	16 ± 0.5	46 ± 1.6	1.9 ± 0.1	1.2 ± 0.1	4.4 ± 0.2	28 ± 1.2

Supplementary Table 2. Comparison of electrochemical CO_2-to-methane with other recent reports.

Catalysts	Potential (V vs RHE)	$J_{partial}$ to CH_4 (mA cm^{-2})	FE (%)	EE (%)	CO_2-to-CH_4 conversion rate (μmol cm^{-2} s^{-1})	Reference
CoO-2.5nm/Cu/PTFE	-1.1	135	60	27	0.17	This work
Cu (75% CO_2)	-1.0	108	48	23	0.14	*J. Am. Chem. Soc.* **142**, 3525 (2020)
$Cu_{68}Ag_{32}$ nanowire	-1.2	50	60	26	0.065	*J. Am. Chem. Soc.* **142**, 12119 (2020)
Cu/CeO_2	-1.8	34.8	55	19	0.045	*ACS Catal.* **8**, 7113 (2018)
Single-atom Zn	-1.8	31.8	85	30	0.041	*J. Am. Chem. Soc.* **142**, 12563 (2020)
Cu	-1.1	27.1	57	26	0.035	*J. Am. Chem. Soc.* **136**, 6978 (2014)
Cu clusters/DRC	-1.0	18.0	81.7	39	0.023	*Angew. Chem. Int. Ed.* (2020) DOI: 10.1002/anie.202009277
Twinned Cu nanowires	-1.3	7.5	55	23	0.0097	*Nano Lett.* **17**, 1312 (2017)
Covalent triazine framework	-0.9	0.3	92	46	0.0004	*Angew. Chem. Int. Ed.* **57**, 13120 (2018)

Supplementary Table 3. FEs to various CO_2RR products on different electrodes as functions of cluster size and loading content.

Notation	J (mA cm^{-2})	CH_4 FE (%)	H_2 FE (%)	C_2H_4 FE (%)	CO FE (%)	Formate FE (%)	Acetate FE (%)	EtOH FE (%)
Co-1.5-1*	225	5.1 ± 0.2	55 ± 1.9	11 ± 0.5	0.7 ± 0.1	12 ± 0.3	5.9 ± 0.1	8.2 ± 0.3
Co-1.5-5	225	26 ± 1.0	61 ± 2.8	3.9 ± 0.2	0.4 ± 0.1	5.1 ± 0.2	1.7 ± 0.1	1.5 ± 0.1
Co-1.5-10	225	18 ± 0.9	59 ± 2.4	8.1 ± 0.2	0.4 ± 0.1	10 ± 0.3	2.4 ± 0.1	3.3 ± 0.2
Co-1.5-20	225	12 ± 0.1	84 ± 4.0	0.5 ± 0.1	0.2 ± 0.1	2.2 ± 0.1	0.1 ± 0.01	0.1 ± 0.01
Co-1.5-30	225	0.4 ± 0.1	99 ± 4.5	0.1 ± 0.01	0.1 ± 0.01	0.1 ± 0.01	0.1 ± 0.01	0.1 ± 0.01
Co-2.1-1	225	6.0 ± 0.2	13 ± 0.4	45 ± 2.0	1.2 ± 0.1	20 ± 0.4	5.5 ± 0.2	8.9 ± 0.4
Co-2.1-5	225	42 ± 1.5	35 ± 1.2	12 ± 0.5	1.0 ± 0.1	6.5 ± 0.2	1.2 ± 0.1	1.5 ± 0.1
Co-2.1-10	225	48 ± 2.8	42 ± 2.1	5.0 ± 0.1	1.0 ± 0.1	2.3 ± 0.1	0.5 ± 0.1	0.2 ± 0.1
Co-2.1-20	225	18 ± 0.4	77 ± 3.0	1.0 ± 0.1	0.2 ± 0.1	1.7 ± 0.1	0.2 ± 0.01	0.2 ± 0.1
Co-2.1-30	225	0.5 ± 0.1	98 ± 4.5	0.1 ± 0.01	0.1 ± 0.01	0.8 ± 0.1	0.1 ± 0.01	0.1 ± 0.01
Co-2.5-1	225	7.2 ± 0.2	6.0 ± 0.3	44 ± 1.9	1.3 ± 0.1	18 ± 0.5	10 ± 0.4	15 ± 0.5
Co-2.5-5	225	45 ± 1.8	19 ± 0.5	18 ± 0.9	1.2 ± 0.1	10 ± 0.4	3.6 ± 0.1	4.1 ± 0.4
Co-2.5-10	225	60 ± 2.2	28 ± 1.2	2.6 ± 0.1	1.1 ± 0.1	3.7 ± 0.2	1.9 ± 0.1	3.5 ± 0.1
Co-2.5-20	225	31 ± 0.8	60 ± 2.8	0.9 ± 0.2	0.5 ± 0.1	2.7 ± 0.1	2.0 ± 0.1	2.5 ± 0.2
Co-2.5-30	225	0.7 ± 0.1	95 ± 4.4	0.1 ± 0.01	0.1 ± 0.01	1.5 ± 0.1	1.1 ± 0.1	0.8 ± 0.1
Co-2.8-1	225	8.1 ± 0.3	9.2 ± 0.4	42 ± 1.5	1.1 ± 0.1	18 ± 0.5	9.1 ± 0.2	8.5 ± 0.5
Co-2.8-5	225	31 ± 1.1	15 ± 0.5	25 ± 1.2	1.0 ± 0.1	15 ± 0.3	6.5 ± 0.2	6.2 ± 0.4
Co-2.8-10	225	36 ± 1.0	33 ± 1.6	20 ± 1.2	0.9 ± 0.1	6.0 ± 0.2	1.1 ± 0.1	2.1 ± 0.1
Co-2.8-20	225	15 ± 0.2	75 ± 3.8	2.5 ± 0.1	0.5 ± 0.1	5.1 ± 0.2	0.6 ± 0.1	1.1 ± 0.1
Co-2.8-30	225	0.5 ± 0.1	96 ± 4.1	0.2 ± 0.1	0.5 ± 0.1	3.1 ± 0.1	0.2 ± 0.01	0.1 ± 0.01
Metal-Co-1	225	0.4 ± 0.1	100 ± 4.5	0.2 ± 0.1	0.1 ± 0.01	0.1 ± 0.01	0.1 ± 0.01	0.1 ± 0.01
Metal-Co-5	225	0.5 ± 0.1	99 ± 4.1	0.1 ± 0.01	0.1 ± 0.01	0.1 ± 0.01	0.1 ± 0.01	0.1 ± 0.01
Metal-Co-10	225	0.6 ± 0.1	101 ± 5.2	0.2 ± 0.1	0.1 ± 0.01	0.1 ± 0.01	0.1 ± 0.01	0.1 ± 0.01
Metal-Co-20	225	0.6 ± 0.1	98 ± 4.0	0.1 ± 0.01	0.1 ± 0.01	0.1 ± 0.01	0.1 ± 0.01	0.1 ± 0.01
Metal-Co-30	225	0.4 ± 0.1	100 ± 5.0	0.1 ± 0.01	0.1 ± 0.01	0.1 ± 0.01	0.1 ± 0.01	0.1 ± 0.01

* In the notation of "Co-X-Y", X is the mean size (nm) and Y is the loading content (ng cm^{-2}).

Supplementary Table 4. EXAFS parameters of the CoO-2.5nm/Cu/PTFE catalyst in *operando* XAS analysis*.

Notation	Shell	Coordination number	Bond length (Å)	ΔE_0 (eV)	$\Delta\sigma^2$
Initial	Co-O	4.5	2.0	2.3	0.093
Operando (at 225 mA cm^{-2})	Co-O	4.5	2.0	3.0	0.069

*ΔE_0, inner potential correction to account for the difference in the inner potential between the sample and the reference compound; $\Delta\sigma^2$, change in the Debye-Waller factor value relative to the Debye-Waller factor of the reference compound. Error bounds (accuracies) that characterize the structural parameters obtained by EXAFS spectroscopy were estimated as coordination numbers, ±20%; bond length, ±1%; ΔE_0, ±20%; $\Delta\sigma^2$, ±20%.

Supplementary Table 5. FEs to various CO_2RR products on different clusters with a ligand concentration of 4 mg ml^{-1} in synthesis and a loading content of 10 ng cm^{-2} on Cu/PTFE electrodes as a function of working current density.

Notation	J (mA cm^{-2})	CH_4 FE (%)	H_2 FE (%)	C_2H_4 FE (%)	CO FE (%)	Formate FE (%)	Acetate FE (%)	EtOH FE (%)
PdO/ Cu/PTFE	100	5.0 ± 0.2	26 ± 1.1	19 ± 1.0	3.9 ± 0.1	8.5 ± 0.3	14 ± 0.5	18 ± 0.7
	150	14 ± 0.5	31 ± 1.2	14 ± 0.6	3.3 ± 0.1	10 ± 0.4	10 ± 0.3	13 ± 0.7
	200	29 ± 0.9	35 ± 1.5	6.4 ± 0.3	1.8 ± 0.1	11 ± 0.4	5.5 ± 0.2	6.4 ± 0.4
	225	47 ± 1.1	35 ± 1.4	2.6 ± 0.1	1.0 ± 0.1	11 ± 0.4	1.8 ± 0.1	2.2 ± 0.1
	250	22 ± 1.0	52 ± 2.0	2.6 ± 0.1	0.3 ± 0.1	16 ± 0.5	1.2 ± 0.1	1.5 ± 0.1
NiO/ Cu/PTFE	100	9.3 ± 0.3	18 ± 0.4	25 ± 1.1	4.2 ± 0.2	5.3 ± 0.2	11 ± 0.4	19 ± 0.5
	150	18 ± 0.8	20 ± 0.5	17 ± 0.7	3.6 ± 0.1	6.1 ± 0.3	9.4 ± 0.4	14 ± 0.4
	200	35 ± 1.5	23 ± 1.4	8.1 ± 0.3	2 ± 0.1	6.9 ± 0.3	3.2 ± 0.1	8 ± 0.5
	225	50 ± 2.1	28 ± 1.0	8.3 ± 0.1	1.0 ± 0.1	8.2 ± 0.2	1.5 ± 0.1	2.8 ± 0.1
	250	30 ± 1.3	39 ± 2.2	3.2 ± 0.1	0.5 ± 0.1	12 ± 0.5	2.1 ± 0.1	2.1 ± 0.1
WO_3/ Cu/PTFE	100	3.4 ± 0.2	17 ± 0.5	35 ± 1.2	4.6 ± 0.2	0.8 ± 0.1	12 ± 0.6	26 ± 0.8
	150	5.7 ± 0.2	20 ± 0.7	30 ± 1.0	4 ± 0.2	1.7 ± 0.1	15 ± 0.6	19 ± 0.5
	200	26 ± 0.8	24 ± 1.1	20 ± 1.0	2.5 ± 0.1	2.4 ± 0.2	8.6 ± 0.4	13 ± 0.4
	225	38 ± 1.3	27 ± 1.3	20 ± 0.2	1.1 ± 0.1	3.0 ± 0.2	4.2 ± 0.1	5.1 ± 0.2
	250	15 ± 0.7	40 ±2.0	16 ± 0.5	1 ± 0.1	18 ± 0.6	1.4 ± 0.1	3.5 ± 0.2
MoO_3/ Cu/PTFE	100	2.1 ± 0.1	16 ± 0.6	37 ± 1.6	4.8 ± 0.2	0.5 ± 0.1	16 ± 0.5	24 ± 1.1
	150	5.2 ± 0.2	18 ± 0.6	35 ± 1.7	4.1 ± 0.2	0.7 ± 0.1	15 ± 0.5	17 ± 1.0
	200	24 ± 1.0	23 ± 0.9	22 ± 0.9	2.5 ± 0.1	1.5 ± 0.1	10 ± 0.3	12 ± 0.5
	225	36 ± 1.9	25 ± 1.0	22 ± 0.5	1.5 ± 0.1	2.2 ± 0.2	5.1 ± 0.2	6.6 ± 0.2
	250	14 ± 0.6	40 ± 1.8	17 ± 0.4	1.5 ± 0.1	15 ± 0.5	3.3 ± 0.2	3.8 ± 0.2

Supplementary Table 6. FEs of different clusters with different ligand concentrations in synthesis and a loading content of 10 ng cm^{-2} on Cu/PTFE under current density of 225 mA cm^{-2} in a flow cell using 1 M $KHCO_3$ electrolyte.

Cluster	Ligand concentration (mg ml^{-1})	J (mA cm^{-2})	CH_4 FE (%)	H_2 FE (%)	C_2H_4 FE (%)	CO FE (%)	Formate FE (%)	Acetate FE (%)	EtOH FE (%)
PdO	16	225	12 ± 0.5	65 ± 3.0	3.0 ± 0.2	0.1 ± 0.01	9.0 ± 0.2	3.3 ± 0.2	2.5 ± 0.1
	8	225	24 ± 1.4	55 ± 2.9	3.3 ± 0.2	0.6 ± 0.1	9.3 ± 0.3	2.8 ± 0.2	2.6 ± 0.2
	4	225	47 ± 1.1	35 ± 1.4	2.6 ± 0.1	1.0 ± 0.1	11 ± 0.4	1.8 ± 0.1	2.2 ± 0.1
	2	225	30 ± 1.0	52 ± 2.5	14 ± 0.9	0.2 ± 0.1	11 ± 0.5	0.8 ± 0.1	1.2 ± 0.1
	0	225	0.5 ± 0.1	105 ± 1.2	0.2 ± 0.1	0.1 ± 0.01	0.1 ± 0.01	0.1 ± 0.01	0.1 ± 0.01
CoO	16	225	18 ± 0.9	59 ± 2.4	8.1 ± 0.2	0.4 ± 0.1	10 ± 0.3	2.4 ± 0.1	3.3 ± 0.2
	8	225	48 ± 2.8	42 ± 2.1	5.0 ± 0.1	1.0 ± 0.1	2.3 ± 0.1	0.5 ± 0.1	0.2 ± 0.1
	4	225	60 ± 2.2	28 ± 1.2	2.6 ± 0.1	1.1 ± 0.1	3.7 ± 0.2	1.9 ± 0.1	3.5 ± 0.1
	2	225	36 ± 1.0	33 ± 1.6	20 ± 1.2	0.9 ± 0.1	6.0 ± 0.2	1.1 ± 0.1	2.1 ± 0.1
	0	225	0.6 ± 0.1	101 ± 5.2	0.2 ± 0.1	0.1 ± 0.01	0.1 ± 0.01	0.1 ± 0.01	0.1 ± 0.01
NiO	16	225	18 ± 1.1	58 ± 3.0	3.7 ± 0.6	0.7 ± 0.1	11 ± 0.5	1.4 ± 0.2	0.5 ± 0.1
	8	225	26 ± 1.3	42 ± 1.3	4.5 ± 0.7	1.0 ± 0.1	10 ± 0.4	1.5 ± 0.2	2.2 ± 0.1
	4	225	50 ± 2.1	28 ± 1.0	8.3 ± 0.1	1.0 ± 0.1	8.2 ± 0.2	1.5 ± 0.1	2.8 ± 0.1
	2	225	31 ± 1.7	32 ± 2.6	20 ± 1.2	0.7 ± 0.1	7.5 ± 0.1	1.1 ± 0.1	1.1 ± 0.1
	0	225	0.3 ± 0.1	98 ± 3.5	0.1 ± 0.01	0.1 ± 0.01	0.1 ± 0.01	0.1 ± 0.01	0.1 ± 0.01
WO_3	16	225	12 ± 0.4	55 ± 2.5	14.4 ± 0.6	0.4 ± 0.1	12 ± 0.8	2.7 ± 0.2	2.9 ± 0.2
	8	225	19 ± 0.8	38 ± 1.7	18 ± 0.4	1.0 ± 0.1	10 ± 0.7	5.4 ± 0.5	6.7 ± 0.4
	4	225	38 ± 1.3	27 ± 1.3	20 ± 0.2	1.1 ± 0.1	3.0 ± 0.2	4.2 ± 0.1	5.1 ± 0.2
	2	225	26 ± 1.1	33 ± 1.2	28 ± 1.0	1.3 ± 0.2	7.4 ± 0.4	4.9 ± 0.2	6.3 ± 0.2
	0	225	8.0 ± 0.8	31 ± 1.1	40 ± 1.7	1.4 ± 0.1	0.4 ± 0.1	5.7 ± 0.5	14 ± 0.8
MoO_3	16	225	8.5 ± 0.4	49 ± 2.1	21 ± 1.8	1.4 ± 0.2	10 ± 0.2	3.2 ± 0.1	4.0 ± 0.5
	8	225	19 ± 0.6	35 ± 1.8	20 ± 1.1	0.8 ± 0.1	13 ± 0.6	3.8 ± 0.3	4.4 ± 0.4
	4	225	36 ± 1.9	25 ± 1.0	22 ± 0.5	1.5 ± 0.1	2.2 ± 0.2	5.1 ± 0.2	6.6 ± 0.2
	2	225	22 ± 1.7	30 ± 1.7	32 ± 1.8	0.5 ± 0.1	6.5 ± 0.2	4.9 ± 0.4	6.2 ± 0.5
	0	225	5.0 ± 0.2	10.7 ± 0.8	43 ± 2.0	0.9 ± 0.1	18 ± 0.4	10 ± 1.0	10 ± 0.4

Supplementary Table 7. FEs of different clusters with a ligand concentration of 4 mg ml^{-1} in synthesis and different cluster loading contents on Cu/PTFE under current density of 225 mA cm^{-2} in a flow cell using 1 M $KHCO_3$ electrolyte.

Cluster	Loading content (ng cm^{-2})	J (mA cm^{-2})	CH_4 FE (%)	H_2 FE (%)	C_2H_4 FE (%)	CO FE (%)	Formate FE (%)	Acetate FE (%)	EtOH FE (%)
PdO	1	225	4.2 ± 0.2	11 ± 0.2	42 ± 1.5	0.8 ± 0.1	22 ± 0.5	7.9 ± 0.4	8.6 ± 0.4
	5	225	39 ± 1.7	26 ± 1.0	10 ± 0.4	0.9 ± 0.1	13 ± 0.4	3.6 ± 0.1	3.3 ± 0.3
	10	225	47 ± 1.1	35 ± 1.4	2.6 ± 0.1	1.0 ± 0.1	11 ± 0.4	1.8 ± 0.1	2.2 ± 0.1
	20	225	6.5 ± 0.2	89 ± 4.0	0.2 ± 0.1	0.2 ± 0.1	1.2 ± 0.1	0.3 ± 0.1	0.3 ± 0.1
	30	225	0.2 ± 0.1	100 ± 4.0	0.2 ± 0.1	0.1 ± 0.01	0.1 ± 0.01	0.1 ± 0.01	0.1 ± 0.01
CoO	1	225	7.2 ± 0.2	6.0 ± 0.3	44 ± 1.9	1.3 ± 0.1	18 ± 0.5	10 ± 0.4	15 ± 0.5
	5	225	45 ± 1.8	19 ± 0.5	18 ± 0.9	1.2 ± 0.1	10 ± 0.4	3.6 ± 0.1	4.1 ± 0.4
	10	225	60 ± 2.2	28 ± 1.2	2.6 ± 0.1	1.1 ± 0.1	3.7 ± 0.2	1.9 ± 0.1	3.5 ± 0.1
	20	225	31 ± 0.8	60 ± 2.8	0.9 ± 0.2	0.5 ± 0.1	2.7 ± 0.1	2.0 ± 0.1	2.5 ± 0.2
	30	225	0.7 ± 0.1	95 ± 4.4	0.1 ± 0.01	0.1 ± 0.01	1.5 ± 0.1	1.1 ± 0.1	0.8 ± 0.1
NiO	1	225	5.6 ± 0.4	5.3 ± 0.2	44 ± 1.0	1.1 ± 0.1	16 ± 0.4	10 ± 0.3	13 ± 0.5
	5	225	41 ± 1.5	16 ± 0.8	12 ± 0.5	1.0 ± 0.1	13 ± 0.3	4.3 ± 0.3	4.7 ± 0.3
	10	225	50 ± 2.1	28 ± 1.0	8.3 ± 0.1	1.0 ± 0.1	8.2 ± 0.2	1.5 ± 0.1	2.8 ± 0.1
	20	225	22 ± 0.7	57 ± 3.8	0.5 ± 0.1	0.3 ± 0.1	1.6 ± 0.1	0.3 ± 0.1	0.5 ± 0.2
	30	225	0.5 ± 0.1	93 ± 4.5	0.3 ± 0.1	0.1 ± 0.01	0.1 ± 0.01	0.1 ± 0.01	0.1 ± 0.01
WO_3	1	225	3.5 ± 0.1	7.4 ± 0.3	46 ± 1.6	2.8 ± 0.1	0.8 ± 0.1	12 ± 0.4	25 ± 1.5
	5	225	28 ± 0.6	19 ± 0.6	25 ± 0.7	1.7 ± 0.1	3.4 ± 0.2	6.9 ± 0.1	12 ± 1.0
	10	225	38 ± 1.3	27 ± 1.3	20 ± 0.2	1.1 ± 0.1	3.0 ± 0.2	4.2 ± 0.1	5.1 ± 0.2
	20	225	4.9 ± 0.4	75 ± 3.7	2.3 ± 0.1	0.7 ± 0.1	2.9 ± 0.1	1.2 ± 0.1	2.7 ± 0.2
	30	225	2.4 ± 0.1	89 ± 4.0	1.7 ± 0.1	0.1 ± 0.01	4.5 ± 0.1	0.4 ± 0.1	0.9 ± 0.1
MoO_3	1	225	2.8 ± 0.2	7.5 ± 0.3	48 ± 2.0	2.5 ± 0.1	1 ± 0.1	11 ± 0.4	24 ± 1.0
	5	225	20 ± 0.8	16 ± 0.7	33 ± 0.9	1.8 ± 0.1	4.6 ± 0.4	7.3 ± 0.3	8.3 ± 0.3
	10	225	36 ± 1.9	25 ± 1.0	22 ± 0.5	1.5 ± 0.1	2.2 ± 0.2	5.1 ± 0.2	6.6 ± 0.2
	20	225	4.1 ± 0.1	71 ± 3.0	3.6 ± 0.2	1.0 ± 0.1	2.4 ± 0.6	1.7 ± 0.	4.2 ± 0.2
	30	225	2.3 ± 0.1	84 ± 4.5	1.8 ± 0.1	0.5 ± 0.1	5.4 ± 0.4	1.1 ± 0.1	1.2 ± 0.1

Supplementary Table 8. FEs to various CO_2RR products on different electrodes as functions of the oxidized clusters with best CH_4 FEs.

Notation	J (mA cm^{-2})	CH_4 FE (%)	H_2 FE (%)	C_2H_4 FE (%)	CO FE (%)	Formate FE (%)	Acetate FE (%)	EtOH FE (%)
PdO	225	47 ± 1.1	35 ± 1.4	2.6 ± 0.1	1.0 ± 0.1	11 ± 0.4	1.8 ± 0.1	2.2 ± 0.1
CoO	225	60 ± 2.2	28 ± 1.2	2.6 ± 0.1	1.1 ± 0.1	3.7 ± 0.2	1.9 ± 0.1	3.5 ± 0.1
NiO	225	50 ± 2.1	28 ± 1.0	8.3 ± 0.1	1.0 ± 0.1	8.2 ± 0.2	1.5 ± 0.1	2.8 ± 0.1
WO_3	225	38 ± 1.3	27 ± 1.3	20 ± 0.2	1.1 ± 0.1	3.0 ± 0.2	4.2 ± 0.1	5.1 ± 0.2
MoO_3	225	36 ± 1.9	25 ± 1.0	22 ± 0.5	1.5 ± 0.1	2.2 ± 0.2	5.1 ± 0.2	6.6 ± 0.2

Supplementary Note 1. The cathodic energy efficiency.

By neglecting the overpotential of oxygen evolution reaction, the cathodic energy efficiency is calculated using the following Equations (1-2):

$$Cathodic\ energy\ efficiency = Cathodic\ voltage\ efficiency \times FE \quad (1)$$

$$Cathodic\ voltage\ efficiency = \frac{1.23 - E_{CH_4}}{1.23 - E_{Applied}} \quad (2)$$

where the $E_{Applied}$ is the applied potential in the experiment, FE is the measured CH_4 FE (%), and E_{CH_4}= 0.17 V vs. RHE for CO_2RR.

Supplementary Note 2. The loading coverage.

We estimate that in the case of the best catalyst (CoO-2.5nm/Cu/PTFE), the coverage of ligand-stabilized CoO clusters on the Cu substrate is 1%, assuming that all CoO clusters are spherical. The following Equations (3-6) are used to estimate the coverage:

$$Coverage\ of\ CoO\ clusters\ on\ Cu\ substrate = \frac{Area\ of\ CoO\ clusters}{Area\ of\ Cu\ substrate} \times 100\% \quad (3)$$

$$Area\ of\ CoO\ clusters = Number\ of\ CoO\ clusters \times \pi \times \left(\frac{Diameter\ of\ CoO\ clusters}{2}\right)^2 \quad (4)$$

$$Number\ of\ CoO\ clusters = \frac{Loading\ content\ of\ CoO\ clusters}{Density\ of\ CoO \times Volume\ of\ one\ CoO\ cluster} \quad (5)$$

$$Volume\ of\ one\ CoO\ cluster = \frac{4}{3} \times \pi \times \left(\frac{Diameter\ of\ CoO\ clusters}{2}\right)^3 \quad (6)$$

in which the diameter and the loading content of CoO clusters are 2.5 nm and 10 ng, respectively, and the area of Cu substrate is 1 cm^2 for the catalyst with the best CH_4 FE of 60%.

Supplementary Note 3 | Reaction energy calculations.

The *H adsorption energies were calculated along the Equation (7):

$$\Delta E_{*H}^{ads} = E_{*H} - E_{*} - E_{H} \tag{7}$$

where E_{*H} is the *H adsorbed on catalyst; E_{*} is the total energy of catalyst; E_{H} is the energy of proton, which was referred from Nørskov's computational hydrogen electrode theory (CHE).

For the hydrogenation of *CO intermediate, we considered the following two Equations (8-9) to achieve this one-electron step simulation when using the *H transfer mechanism:

$$H_3O^+ + e^- \rightarrow H_2O + {}^*H \tag{8}$$

$${}^*H + {}^*CO \rightarrow {}^*CHO \tag{9}$$

The reaction energy of (9) was calculated by the Equation (10):

$$\Delta E = E_{*CHO} - E_{*H+*CO} \tag{10}$$

where E_{*H+*CO} and E_{*CHO} are the total energies of {*H and *CO}, and *CHO, adsorbed on the catalyst, respectively. The hydrogenation energies for metal/metal oxide decorated Cu shown in Fig. 1a were then obtained by combining the value of energy in Equations (7) and (10).

The hydrogenation reaction energy for the *Heyrovsky* mechanism was calculated on pure Cu and is described by the Equation (11):

$$H_3O^+ + e^- + {}^*CO \rightarrow H_2O + {}^*CHO \tag{11}$$

The reaction energy of Equation (11) was calculated by the Equation (12):

$$\Delta E = E_{*CHO} + E_{H_2O} - E_{*CO} - E_{H_3O^+} \tag{12}$$

where E_{*CO} is the total energy of *CO adsorbed on catalyst. $E_{H_3O^+}$ is obtained from proton energy of CHE.
